# Comparison of symptomatic and asymptomatic infections due to severe acute respiratory coronavirus virus 2 (SARS-CoV-2) in San Francisco long-term care facilities

**DOI:** 10.1017/ice.2020.1371

**Published:** 2020-12-14

**Authors:** Janice K. Louie, Juliet E. Stoltey, Hyman M. Scott, Stephanie Trammell, Ejovwoke Ememu, Michael C. Samuel, Tomas J. Aragon, Godfred Masinde

**Affiliations:** 1 San Francisco Department of Public Health, San Francisco, California; 2 University of California, San Francisco, San Francisco, California; 3 University of California, Berkeley, Berkeley, California

*To the Editor—*Severe acute respiratory coronavirus virus 2 (SARS-CoV-2), the virus that causes coronavirus disease 2019 (COVID-19) infection, has contributed to substantial mortality in older populations in long-term care facilities (LTCFs). With high prevalence of asymptomatic infection reported in both healthcare workers (HCWs) and residents, efforts to prevent the introduction and transmission of COVID-19 in these high-risk settings has led to costly resource and labor-intensive national recommendations.^1–5^

Accordingly, in March 2020 the San Francisco Department of Public Health implemented stringent recommendations in skilled nursing facilities (SNFs) and assisted living facilities (ALFs) according to Centers for Disease Control and Prevention COVID-19 recommendations.^5^ These included restrictions of visitors and communal activities, rigorous infection control practices, universal masking, and enhanced surveillance for and testing of suspect cases. From March 12 to May 15, 2020, the suspect case definition included fever (≥37.8°C), cough, or shortness of breath. After May 15, additional symptoms included fatigue, myalgias, headache, new loss of taste or smell, sore throat, congestion, nausea, vomiting, diarrhea or altered mental status. Once COVID-19 cases were identified, outbreak response included contact investigation and facility-wide testing, cohorting of infected from noninfected residents, and home isolation of infected HCWs. On May 7, periodic facility-wide surveillance testing regardless of presence of symptoms was implemented in SNFs.

Laboratory testing with reverse-transcription polymerase chain reaction (RT-PCR) was performed at either hospital or commercial laboratories or the San Francisco Public Health Laboratory (SFPHL). Persons with absence of the aforementioned symptoms at time of specimen collection were defined as asymptomatic. We used the Mann-Whitney U test to compare the cycle threshold (Ct) values of symptomatic and asymptomatic persons, and the Pearson correlation coefficient to measure the association between Ct values and age.

These activities were public health surveillance, and not research; therefore, institutional review board review was not obtained.

## Results

From March 30 to June 15, 2020, we identified 5 COVID-19 outbreaks in 4 SNFs and 1 elderly ALF. In total, 543 persons were tested and 184 (33.9%) were SARS-CoV-2 positive (Table 1). Of these 184 SARS-CoV-2–positive persons, 63 (34.2%) were symptomatic (including 31 HCWS and 32 residents) and 121 (65.7%) were asymptomatic (including 45 HCWs and 76 residents). The median age for a COVID-19-infected person was 71.0 years (range, 21–99).

**Table 1.** Characteristics of SARS-CoV-2-Infected Symptomatic and Asymptomatic Persons in San Francisco Long-Term Care Facilities

Characteristic	Total, No. (%)	Symptomatic, No. (%) N = 63 (34.2)		Asymptomatic, No. (%) N = 121 (65.7)	
		HCW	Residents	HCW	Residents
SARS-CoV-2 infected	184	31 (49.2)	32 (50.8)	45 (37.2)	76 (62.8)
Age, median y (range)	71.0 (21–99)	54.0 (23–77)	85.0 (57–98)	48.0 (21–72)	79.0 (47–99)
Hospitalized^a,b^	30/183 (16.4)	0	24 (75.0)	0	6/75 (8.0)
Died^b^	14 (7.6)	0	10 (31.3)	0	4 (5.3)
Tested at SFPHL^c^	106 (57.6)	11 (17.4)		95 (78.5)	
RT-PCR cycle threshold, median (range)^d^	16.4 (3.0–29.7)	11.5 (3.0–21.8)		16.7 (3.3–29.7)	

Note. HCW, healthcare worker; SFPHL, San Francisco Public Health Laboratory; RT-PCR, reverse-transcription polymerase chain reaction.

a Includes patients with known information only; when incomplete information available, the denominator is noted.

b Hospitalization or death due to COVID-19 as recorded in the medical record or death certificate, as appropriate.

c Testing of oropharyngeal or nasopharyngeal swabs was performed with the RealTime SARS CoV-2 molecular amplification assay using the Abbott m2000 platform. Cycle threshold values below 30.5 indicate a positive result.

d Cycle threshold values for symptomatic persons were excluded if the time from symptom onset to specimen collection was ≥10 days or if persons tested positive prior to symptom onset.

No COVID-19–infected HCWs were hospitalized or died. Of 107 COVID-19–infected residents with available information, 30 (28%) were hospitalized (including 24 symptomatic and 6 asymptomatic residents) and 14 (13.0%) died (including 10 symptomatic residents and 4 without documented symptoms prior to being found unconscious).

Of 184 persons with positive SARS-CoV-2 tests, 106 (57.6%) were tested at the SFPHL; 11 (10.4%) were symptomatic (including 3 HCWs and 8 residents); and 95 (89.6%) were asymptomatic (including 30 HCWs and 65 residents). The overall median Ct value was 16.4 (range, 3.0–29.7). The median Ct values for symptomatic and asymptomatic persons were 11.5 (range, 3.0–21.8) and 16.7 (range, 3.3–29.7), respectively. There was no significant difference when comparing the median Ct values of symptomatic and asymptomatic persons, and no significant correlation between Ct value and age (Pearson correlation coefficient -0.06; 95% confidence interval [CI], −0.25 to 0.13; *P* > .05).

## Discussion

Mass testing in LTCFs revealed that >65% of SARS-CoV-2–infected HCWs and residents were asymptomatic. Cycle threshold values, which may be an indirect proxy for viral load, were comparable in both asymptomatic and symptomatic populations.^6^ Although the high prevalence of asymptomatic infection in LTCFs has been well documented, descriptions of associated Ct values are limited. In 2 SNF outbreaks in Washington where >50% of infected residents were asymptomatic, Ct values were comparable in asymptomatic, presymptomatic, and symptomatic persons.^1,2^ Other studies, including a study of young adults in 2 Korean outpatient settings and a study from public health surveillance testing of mostly adults in England, identified no significant difference in Ct values in asymptomatic compared to symptomatic populations.^7–9^ These findings support biologic plausibility for the potential of high risk of asymptomatic transmission.

Similar to others, in our study, a larger proportion of symptomatic SARS-CoV-2–infected LTCF residents required hospitalization or died compared to their asymptomatic counterparts.^1,2,4^ Using symptoms to ascertain SARS-CoV-2 infection in older adults can be challenging, especially in those with dementia who may present atypically.^10^ SARS-CoV-2–infected LTCF residents should be closely monitored for development of new or acute worsening of chronic symptoms, which may portend a high risk for clinical deterioration and death.

This study had several limitations. Many more asymptomatic persons were tested at the SFPHL compared to symptomatic HCWs and residents, as symptomatic persons were more likely to be referred to clinics or emergency rooms for clinical assessment and testing. During the early part of surveillance, facilities monitored for fever or respiratory symptoms but did not systematically ask about other symptoms (eg, rhinorrhea or diarrhea); therefore, some cases identified as asymptomatic may have had atypical or nonrespiratory COVID-19 symptoms. The presence of symptoms was ascertained at time of specimen collection. Although LTCFs were instructed to report any new symptoms in SARS-CoV-2–infected persons after specimen collection, some presymptomatic infections may have been missed, although this is thought to be a small number.

In summary, in this study, a large proportion of LTCF HCWs and residents had asymptomatic COVID-19 infection, and Ct values suggested levels of viral shedding comparable to those with symptomatic infection. Our findings emphasize that current extensive recommendations for surveillance and management of SARS-CoV-2 transmission and COVID-19 in high-risk LTCF settings are warranted, including rigorous employment of isolation and infection control measures, universal use of personal protective equipment, and both symptom-based and periodic surveillance facility-wide testing to identify both symptomatic and asymptomatic infected persons.
